# HO-3867 Induces ROS-Dependent Stress Response and Apoptotic Cell Death in *Leishmania donovani*

**DOI:** 10.3389/fcimb.2021.774899

**Published:** 2021-12-03

**Authors:** Amrita Das, Mohd. Kamran, Nahid Ali

**Affiliations:** Infectious Diseases and Immunology Division, Council of Scientific and Industrial Research (CSIR)-Indian Institute of Chemical Biology, Kolkata, India

**Keywords:** liposome, stress, *Leishmania donovani*, HO-3867, apoptosis

## Abstract

Lack of vaccine and increasing chemotherapeutic toxicities currently necessitate the development of effective and safe drugs against various forms of leishmaniases. We characterized the cellular stress induced by a novel curcumin analogue, HO-3867, encapsulated within the phosphatidylcholine-stearylamine (PC-SA) liposome for the first time against *Leishmania*. The liposomal formulation of HO-3867 (i.e., PC-SA/HO-3867) initiated oxidative stress-induced apoptosis in *L. donovani*, revealed by altered cell morphology, phosphatidylserine externalization, mitochondrial depolarization, intracellular lipid accumulation, and cell cycle arrest in promastigotes. Liposomal HO-3867 was observed to be a strong apoptosis inducer in *L. donovani* and *L. major* in a dose-dependent manner, yet completely safe for normal murine macrophages. Moreover, PC-SA/HO-3867 treatment induced *L. donovani* metacaspase and PARP1 activation along with downregulation of the Sir2 gene. PC-SA/HO-3867 arrested intracellular *L. donovani* amastigote burden *in vitro*, with reactive oxygen species (ROS) and nitric oxide (NO)-mediated parasite killing. These data suggest that liposomal HO-3867 represents a highly promising and non-toxic nanoparticle-based therapeutic platform against leishmaniasis inspiring further preclinical developments.

## Introduction

Visceral leishmaniasis (VL) or kala-azar is one of the deadly systemic infections caused by *Leishmania donovani*/*Leishmania infantum* with estimated 200,000–400,000 new cases and more than 20,000–30,000 deaths per year [http://www.who.int/mediacentre/factsheets/fs375/en/]. Emergence of resistant parasites, drug-related toxicities, and HIV coinfection has further complicated the scenario of VL treatment in endemic regions (Singh et al., 2016; Alves et al., 2018). Thus, there is an unmet challenge to develop safe and alternative drugs to treat this fatal infection. HO-3867, a curcumin analogue belonging to class diarylidenylpiperidone (DAP), is a robust STAT3 inhibitor inducing reactive oxygen species (ROS), caspase-3, and PARP1-mediated apoptosis in cancer cells (Selvendiran et al., 2010; Tierney et al., 2012; Rath et al., 2014; Madan et al., 2018; Mast et al., 2019; Li et al., 2020). However, its potency has never been evaluated against any kinetoplastid parasites so far. Despite their multiple benefits, curcuminoids have limited clinical applications due to low bioavailability (Mohammed et al., 2004; Anand et al., 2007; Sinjari et al., 2019). PC-SA liposomes reportedly target surface exposed anionic phosphatidylserine (PS) abundant on majority of cancer cells (De et al., 2018) and *Leishmania* (Banerjee et al., 2008; Wanderley et al., 2020) owing to the direct interaction of this anionic membrane lipid with SA. Herein, we envisioned the potential targeting of *Leishmania* parasites by PC-SA liposomes encapsulating HO-3867 for effective therapy. The enhanced ROS accumulation in PC-SA/HO-3867-treated parasites leads to cell cycle arrest and concurrent depolarization of mitochondrial membrane potential (Zhao et al., 2004) possibly causing release of cytochrome c in the cytoplasm and DNA damage. This indicates involvement of the mitochondria-dependent intrinsic pathway (Kroemer et al., 2007) of cell death in *L. donovani* after PC-SA/HO-3867 incubation, which in turn activates metacaspase and PARP-1 as precursors of apoptosis. The results are further supported by ROS and NO-mediated inhibition of intracellular amastigote multiplication in murine peritoneal MΦ after treatment, which needs further *in vivo* evaluations.

## Method

### Animals and Parasites

Healthy BALB/c mice bred at the animal house facility of the CSIR-Indian Institute of Chemical Biology [approved by the Committee for the purpose of Control and Supervision on Animal Experiments (CPCSEA), Govt. of India, and the Animal Ethics Committee (147/1999/CPSCEA) of CSIR-IICB] were used. *L. donovani* strain AG83 (ATCC^®^ PRA413™) were cultured at 22°C in M199 (Sigma-Aldrich, St. Louis, MO) medium supplemented with 10% heat-inactivated FCS, 2 mM glutamine, penicillin (100 U/ml), and streptomycin (100 µg/ml) (Sigma-Aldrich). Stationary-phase parasites are periodically subcultured to maintain an average density of 2 × 10^6^ cells/ml in M199 medium. *L. major* promastigotes strain 5ASKH (a kind gift from Dr. Subrata Adak, CSIR-IICB) were cultured at 26°C in M199 medium with 10% heat-inactivated FCS, 200 µM adenine, penicillin (100 U/ml), streptomycin (100 µg/ml), and 40 mM HEPES (Dolai et al., 2008).

### Preparation and Characterization of PC-SA/HO-3867 Liposomes

Cationic liposomes were prepared with 20 mg of phosphatidylcholine (PC) (Sigma-Aldrich) with stearylamine (SA) (Fluka) at a 7:2 molar ratio by thin-film rehydration method (De et al., 2018). For drug encapsulation, a methanolic solution of HO-3867 (Cayman Chemicals) (1 mg/ml) was added to the lipid film, dried overnight, and finally dispersed in 1 ml of 0.02 M PBS (pH = 7.4) to form a stock solution of 20 mg/ml with respect to PC. Rhodamine B (RhB)-labeled liposomes were prepared by adding 0.1 mg/ml of RhB (Sigma) in organic phase together with PC and SA followed by lipid film dispersion in 0.02 M PBS containing 5-carboxyfluorescein (0.1 mg/ml).

Particle sizes of PC-SA and PC-SA/HO-3867 were determined by DLS (dynamic light scattering) using Zetasizer Nano ZS (Malvern Instruments) as described previously (De et al., 2018). The morphology of liposomes was examined by atomic forced microscopy (AFM) following standard procedures after placing the sample on a freshly cleaved mica grid (Das et al., 2018).

Measurement of encapsulation efficiency was based on the absorbance at different concentrations of HO-3867 measured at 328 nm using a UV-visible spectrophotometer, calculated from the amount of free HO-3867 present in the unentrapped fraction after ultracentrifugation (100,000× *g* for 1 h, at 4°C) using the formula:


$$\text{Percent encapsulated }=\frac{\left[\text{Total HO}-3867]-[\text{Free HO}-3867\right]}{\text{Total HO}-3867}\times 100\%$$

### Cellular Uptake

Peritoneal macrophages (MΦ) isolated from naïve BALB/c mice are cultured overnight on coverslips (18 mm^2^, 10^6^ MΦ/coverslip) at 37°C with 5% CO_2_ in RPMI medium (Sigma-Aldrich) (Das et al., 2018). Resident MΦs were infected with freshly transformed *L. donovani* promastigotes at a ratio of 1:10 for 4 h at 37°C with 5% CO_2_. To assess the uptake efficacy and intracellular docking of free liposomes, infected and uninfected MΦ were treated with Rhodamine B (RhB) (red)-labeled PC-SA liposomes loaded with 5-carboxyfluorescein dye (green) for 2 h. The cells were next fixed with 4% paraformaldehyde (pH 7.4) and counterstained with DAPI (Invitrogen). The fluorescent signals were captured by a TCS-SP8 (Leica Microsystems, Germany) microscope equipped with Leica LAS X live cell imaging software. The lasers used were 405, 488, and 577 argon lasers for DAPI, 5-carboxyfluorescein, and RhB, respectively.

### Promastigote and Amastigote Inhibitory Assays

To evaluate the effects of HO-3867 on promastigotes, 2 × 10^5^ parasites/ml were treated with graded concentrations of PC-SA liposomes and free and liposomal HO-3867 for 2 h, at 22°C. The untreated and treated parasites were further incubated with 2 mg/ml of MTT [3,-(4,5-dimethylthiazol-2-yl)-2,5-diphenyltetrazolium bromide] (Affymetrix) solution for 2 and 4 h at 37°C as detailed previously (Shadab et al., 2017). The reduced formazan crystals were dissolved in DMSO, and absorbance was measured at 550 nm in a spectrophotometer.

For evaluating inhibitory effects of free and liposomal HO-3867 on intracellular amastigotes, RAW 264.7 cells (2 × 10^5^ cells/coverslip) were infected with log-phase promastigotes of *L. donovani* (in 1:10 ratio) for 4 h in RPMI 1640 medium supplemented with 10% FCS. Following the removal of free promastigotes by vigorous washing with 0.02 M PBS thrice, infected MΦs were incubated without drug for an additional 24 h at 37°C in 5% CO_2_. Next, the infected MΦs were treated with free or liposomal HO-3867 (2, 5, 10, 30, 50, and 100 µg/ml) diluted in fresh medium for 2 h. After the removal of liposomes and drug, the infected MΦs were further kept for 72 h at 37°C in a CO_2_ incubator. After 72 h, the coverslips were washed with 0.02 M PBS and fixed with methanol (Merck) followed by Giemsa staining (1:20 dilution with deionized water, kept for 5–10 min). The number of amastigotes was counted under light microscope for 200 MΦs per sample and expressed as means of three independent experiments. The 50% inhibitory concentration (IC_50_) of free and liposomal drug was calculated for *L. donovani* promastigotes and intracellular amastigotes *via* a non-linear curve.

### Determination of Cell Morphology

*L. donovani* promastigotes treated with IC_50_ concentration of free and liposomal HO-3867 and empty PC-SA liposomes for 2 h at 22°C were harvested, washed in 0.02 M PBS, fixed in 4% paraformaldehyde on 18-mm^2^ glass coverslips, and air dried. Cells were sometimes washed in 0.05 ml Milli-Q water to remove the salt depositions and air dried as mentioned. Contact mode AFM was done by PicoPlus 5500 AFM using a piezoscanner in a maximum range of 100 µm and processed using PicoView 1.8 Advanced Software.

### PS Exposure and TUNEL Assay

For Annexin-V binding, differently treated and untreated 1 × 10^6^ *L. donovani* promastigotes were suspended in 1 ml of 1× Annexin-V binding buffer (BD Biosciences) and incubated for 5 min in the dark, at room temperature. Following addition of 4 µl of propidium iodide (PI) (BD Biosciences), cells were analyzed by flow cytometry.

Terminal deoxyribonucleotidyl transferase (TdT)-mediated dUTP nick-end labeling (TUNEL) assay using an *in-situ* apoptosis detection kit (Takara) was performed to detect fragmented DNA in *L. donovani* promastigotes treated with or without liposomal HO-3867 for 2 h at 22°C, according to the manufacturer’s instructions. Briefly, 1 × 10^6^ cells were fixed in 4% paraformaldehyde (pH = 7.4) for 15 min and permeabilized using 100 µl of permeabilization buffer for 5 min on ice. The cells were resuspended in 50 µl of TUNEL labeling reaction mixture (TdT enzyme + labeling solution) and incubated in a dark-humidified chamber at 37°C for 60 min. Cells treated only with 50 µl of labeling solution (without enzyme) which served as negative control. The reaction was finally terminated by washing with 0.02 M PBS. The green fluorescence of apoptotic cells was analyzed by confocal microscopy at 488 nm.

### Measurement of Mitochondrial Membrane Potential

Briefly, treated and untreated promastigotes (1 × 10^6^ cells) were resuspended in 500 µl of 2 µM JC-1 (Molecular Probes) and incubated for 15 min at 37°C in the dark (De et al., 2018). Subsequently, the cells were washed in 0.02 M PBS and subjected to confocal microscopy following nuclear staining with Hoechst 33342 blue (Molecular Probes) for 5 min. The live cell confocal images were captured with 405 for Hoechst 33342, and 488 and 560 argon lasers for JC-1.

### Caspase-Like Activity and ROS Estimation

*In-vivo* caspase-like activity in *L. donovani* promastigotes was evaluated by flow cytometry using Intracellular Caspase Detection ApoStat Kit (R& D systems) following the manufacturer’s instructions. After liposomal HO-3867 exposure of promastigotes at 22°C, cells were stained directly in culture medium for the last 30 min of treatment at 37°C in a CO_2_ incubator with 1 µl ApoStat/100 µl of cells. Cells were harvested, washed briefly in 0.02 M PBS and analyzed by flow cytometry using BD LSRFortessa.

Intracellular ROS (reactive oxygen species) generation after treatment of promastigotes with free and liposomal HO-3867 was measured using 10 µM of green oxidant-sensitive dye 2′,7′-dichlorodihydrofluorescein diacetate (H_2_DCFDA, Molecular Probes) dye as reported earlier (Shadab et al., 2017; Das et al., 2018). After 30 min of incubation, cells were washed in 0.02 M PBS and finally analyzed by flow cytometry.

### Quantification of Membrane Integrity and Lipid Accumulation

*L. donovani* promastigotes harvested with or without treatment were stained with 10 µg/ml Nile Red (Sigma-Aldrich) for 30 min followed by following nuclear staining with Hoechst 33342 blue (Molecular Probes) for 5 min. Confocal live cell images were captured with 405 for Hoechst 33342, and 470 (for non-polar/neutral lipids) and 546 (for polar lipids) argon lasers for Nile Red (Rosa et al., 2015).

### Real-Time PCR

Real-time PCR analysis for *L. donovani* metacaspase, PARP-1 genes, and SIR2 genes was done with gene-specific primers (Purkait et al., 2012; Shadab et al., 2017). Primers for amplifying metacaspase, Sir2, and PARP1 cDNAs were as follows: LdMetacaspase forward, 5′ AAA CGG GTC GAC ATT AAT GC 3′ and LdMetacaspase reverse 5′ CGA GCA TGA GGA AAA GAT CA3′, LdSir2 forward, 5′ GGTCATCATCATCGGGACAT 3′ and LdSir2 reverse 5′ TCATCAGGAA AGCGGAAGAG 3′, LdPARP1 forward, 5′ TGCCGGAAGGCGGCTCATTC 3′ and LdPARP1 reverse, 5′ CGCAGTGCGTTGCGCATACC 3′, GAPDH forward, 5′ GAAGTACACGGTGGAGGCTG 3′, GAPDH reverse, 5′CGCTGATCACGACCTTCTTC 3′. *L. donovani* cDNAs were amplified using SYBR Green Real-Time Master Mix (Roche). Gene expression levels were determined from Cq values followed by normalization of values of each gene (Livak and Schmittgen, 2001; Schmittgen and Livak, 2008) with expression levels of GAPDH by 2^-ΔΔ^ *^Ct^*. The fold expression was calculated as:


$$\text{Fold expression }=\;2^{-}\text{ΔΔ}Ct$$

### DNA Fragmentation and Cell Cycle Analysis in *L. donovani*

*L. donovani* promastigotes were treated with 10 µg/ml of free and liposomal HO-3867 or empty PC-SA liposomes for 2 h, and genomic DNA was isolated using the Suicide Track ™ DNA Ladder Isolation Kit (Calbiochem) using the manufacturer’s instructions (Shadab et al., 2017). After washing in 70% ethanol, the genomic DNA pellets obtained were finally dispersed in resuspension buffer (10 Mm Tris–HCl, pH = 7.5, 1 Mm EDTA) and visualized with 0.5 µg/ml ethidium bromide (EtBr) staining, under a Bio-Rad Gel Documentation system.

For cell cycle analysis, after for 2 and 6 h of drug treatment, *L. donovani* promastigotes were harvested, washed in 0.02 M PBS, and fixed in 70% ethanol (constituted in PBS) overnight. Cells were stained with FxCycle™ PI/RNase Staining Solution (Molecular Probes) for 30 min at room temperature. The percentage of cells in different cell cycle phases, i.e., G0, G0/G1, S, and G2/M, was gated using BD LSRFortessa and analyzed by BD FACS Scan Software.

### Evaluation of *In Vitro* Macrophage (MΦ) Infection

Peritoneal MΦs collected from naïve BALB/c mice were plated overnight in RPMI 1640 medium on 18-mm^2^ glass coverslips as detailed previously (Das et al., 2018). MΦs were infected *in vitro* with *L. donovani* promastigotes at a 1:10 ratio for 4 h at 37°C in a CO_2_ incubator. After removing free promastigotes by three successive washings with 0.02 M PBS, infected MΦs were incubated without drug for 24 h at 37°C in 5% CO_2_. Next, the infected MΦs were treated with free or liposomal HO-3867 (10 µg/ml) diluted in fresh medium for 2 h. Excess liposomes and drug were removed after 2 h, and the infected MΦs were kept for an additional 48 and 72 h at 37°C in a CO_2_ incubator. Finally, the coverslips were washed with 0.02 M PBS and fixed with methanol (Merck) followed by Giemsa staining. The number of amastigotes was counted under a light microscope for 200 MΦs per sample and expressed as means of three independent experiments. Nitric oxide (NO) production was quantified in culture supernatants of HO-3867 and PC-SA/HO-3867 (10 µg/ml) treated MΦs by Griess Reagent (absorbance 540 nm) (Das et al., 2018). ROS generation in the infected MΦs was assessed by 10 µM H_2_DCFDA using a Synergy H1 (Biotek) microplate reader (excitation/emission = 485 nm/528 nm) (Das et al., 2018).

### Statistical Analysis

GraphPad prism version 6.0 (GraphPad Software) was used for statistical analyses. One-way analysis of variance (ANOVA) and Tukey–Kramer multiple-comparison test were used for comparing between groups. *p* < 0.05 was considered as statistically significant difference for all the experiments.

## Results and Discussion

### Characterization of PC-SA/HO-3867

**Figure 1:**
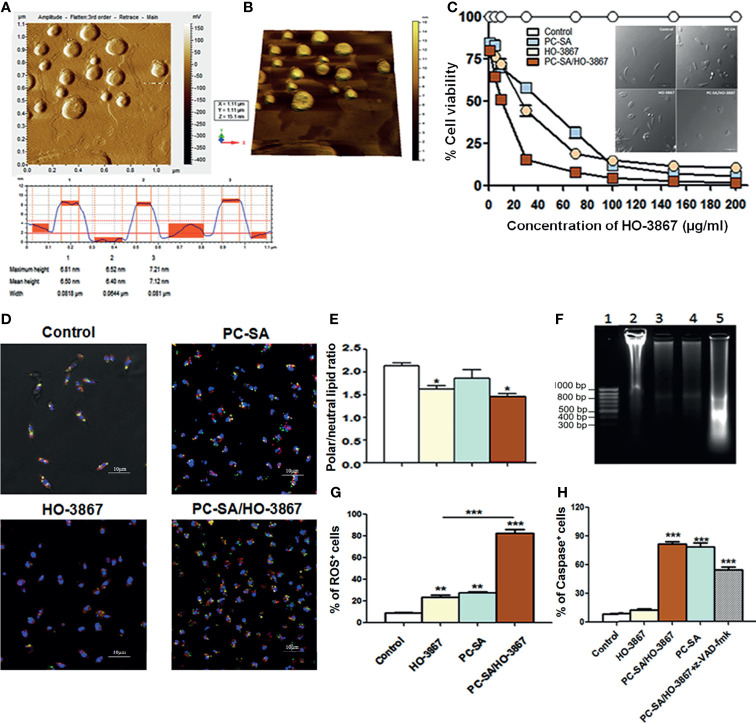
Vesicle morphology and effect of liposomal HO-3867 on *L. donovani* promastigotes 2 h post-treatment. **(A)** Tapping mode AFM images of PC-SA/HO-3867 liposomes. Topography is indicative of the height of the liposomes from the mica base. **(B)** Representative three-dimensional AFM image of PC-SA/HO-3867 liposomes. **(C)** Effect of increasing concentrations of empty liposomes and free and liposomal HO-3867 on viability of log-phase promastigotes estimated by MTT assay. Inset depicts the morphological alterations of control, empty PC-SA liposomes (10 µg/ml w.r.t PC), and drug-treated promastigotes under a phase-contrast microscope. **(D)** Nile Red (NR)-fluorescent quantification of polar (orange-red) and non-polar (yellowish green) lipid accumulation in promastigotes with or without free and liposomal HO-3867 treatment. **(E)** Representative bar diagram showing the ratio of polar/non-polar (i.e., red/green) lipids in differently treated promastigotes. **(F)** DNA fragmentation in promastigotes treated with IC_50_ dose of PC-SA/HO-3867, free HO-3867, and PC-SA liposomes. Lane 1, GeneRuler 50-bp DNA ladder (Thermo); Lanes 2–5, untreated, promastigotes treated with 10 µg/ml of free HO-3867, PC-SA liposomes, and liposomal HO-3867, respectively. **(G)** Intracellular ROS generation in log-phase promastigotes after treatment with free and liposomal HO-3867, by H_2_DCFDA. **(H)** Bar graph showing means of caspase-positive cells in differently treated and untreated parasites, in absence or presence of pan-caspase inhibitor z-VAD-fmk, as analyzed by flow cytometry. Each data represent mean ± S.E. (n = 3) and is one of the three experiments with similar results. *p < 0.05; **p < 0.001; ***p < 0.0001, as assessed by one-way ANOVA and Tukey’s multiple-comparison test.

HO-3867-entrapped PC-SA liposomes were developed by thin-film rehydration method (De et al., 2018). Stearylamine (SA) was incorporated to impart high *Leishmania* targeting ability of these cationic liposomes (Banerjee et al., 2008). The mean particle size, zeta potential, and drug encapsulation efficacy of PC-SA/HO-3867 were 164 ± 15 nm, 46 ± 8.5 mV, and 88.4 ± 5.2%, respectively. The morphology of PC-SA/HO-3867 liposomes as characterized by AFM at 150–300 kHz frequency (Zhao et al., 2004) depicted their smooth spherical structures without visible aggregation (**Figures 1A, B**).

### PC-SA/HO-3867 Induced Cell-Death in *L. donovani* Is Chiefly ROS-Mediated

MTT (Invitrogen) colorimetric assay reveals the significant dose-dependent cytotoxicity of free and liposomal HO-3867 (1–200 µg/ml) against promastigotes (**Figure 1C**) and intracellular amastigotes (**Supplementary Table 1**) of *L. donovani* without affecting normal mammalian cells, even after treatment with 100 µg/ml of PC-SA/HO-3867 (equivalent to PC-SA/HO-3867 containing 215.26 µM of HO-3867 in liposomes). IC_50_ values of free HO-3867 against *L. donovani* and *L. major* were 30 and 33.5 µg/ml, respectively. Much lower IC_50_ values of 10 and 12.5 µg/ml were obtained after exposure to PC-SA/HO-3867 in *L. donovani* and *L. major*, respectively.

Accumulation of excess cellular lipid has long been associated with stress-induced apoptosis (Boren and Brindle, 2012; Rosa et al., 2015). To address the changes in the ratio of polar and non-polar lipids after treatment, lipophilic marker Nile Red (0.1 µg/ml) was used, which shows the characteristic shift from red to green emission in the presence of polar and non-polar lipids, respectively (Greenspan et al., 1985; Rosa et al., 2015). Intense yellowish green (neutral) fluorescent lipid droplets are seen to be accumulated in drug-treated promastigotes compared to prominent orange red punctuations (polar) of membrane lipids seen in control (**Figure 1D**). Also, quantitative analysis of whole cells (ex 514 ± 20/em 550 ± 40 for non-polar or neutral lipids; ex 560 ± 20/em 620 ± 50 for polar lipids) revealed significant accumulation of neutral lipids in drug-treated parasites over controls. The red/green ratio changed from 2.2 ± 0.1 in controls to 1.6 ± 0.14 in HO-3867 and 1.4 ± 0.12 in PC-SA/HO-3867-treated parasites, confirming the accumulation of non-polar/neutral lipids after 2 h (**Figure 1E**). Similar to earlier reports (Kathuria et al., 2014; Garcia et al., 2017), our study shows leishmanicidal activity of HO-3867 with surplus accumulation neutral lipid bodies in the cytoplasm indicating faulty lipid metabolism. We also performed DNA laddering assay (**Figure 1F**), which shows marked genomic DNA fragmentation in HO-3867, PC-SA, and HO-3867/PC-SA-treated *L. donovani* promastigotes compared to untreated controls (showing no such oligonucleosome-sized fragments).

Although sublethal ROS production sustains cellular homeostasis, a drastic ROS upsurge is associated with apoptosis (Basmaciyan et al., 2018). Fluorescent labeling of cells with H_2_DCFDA showed an almost fourfold increase in intracellular ROS (**Figure 1G**) in promastigotes compared to free drugs indicative of imbalanced cellular redox homeostasis and programmed cell death (PCD) in parasites after 2 h. As a key regulator of PCD, the presence of caspase-like proteases was evaluated in untreated and treated *L. donovani* promastigotes (Shadab et al., 2017). A significantly high caspase-like activity of empty PC-SA liposomes and PC-SA/HO-3867 was observed compared to free HO-3867 (**Figure 1H**). To confirm the assay specificity, promastigotes were preincubated for 1 h with z-VAD-fmk, a pan-caspase inhibitor, followed by PC-SA/HO-3867 treatment which showed significant reduction (*p* < 0.01) in absorbance. Increased caspase-like activity in promastigotes by PC-SA/HO-3867 treatment is possibly due to PC-SA liposomes, a known activator of caspase-mediated cell death (De et al., 2018). We also observed involvement of caspase-independent cell death in free HO-3867-treated *L. donovani* promastigotes but caspase-mediated apoptosis after PC-SA and PC-SA/HO-3867 treatment. This is probably due to the synergistic antileishmanial activity of PC-SA liposomes and HO-3867.

### PC-SA/HO3867 Causes Altered Cell Morphology, DNA Fragmentation, and Cell Cycle Arrest in Promastigotes

**Figure 2:**
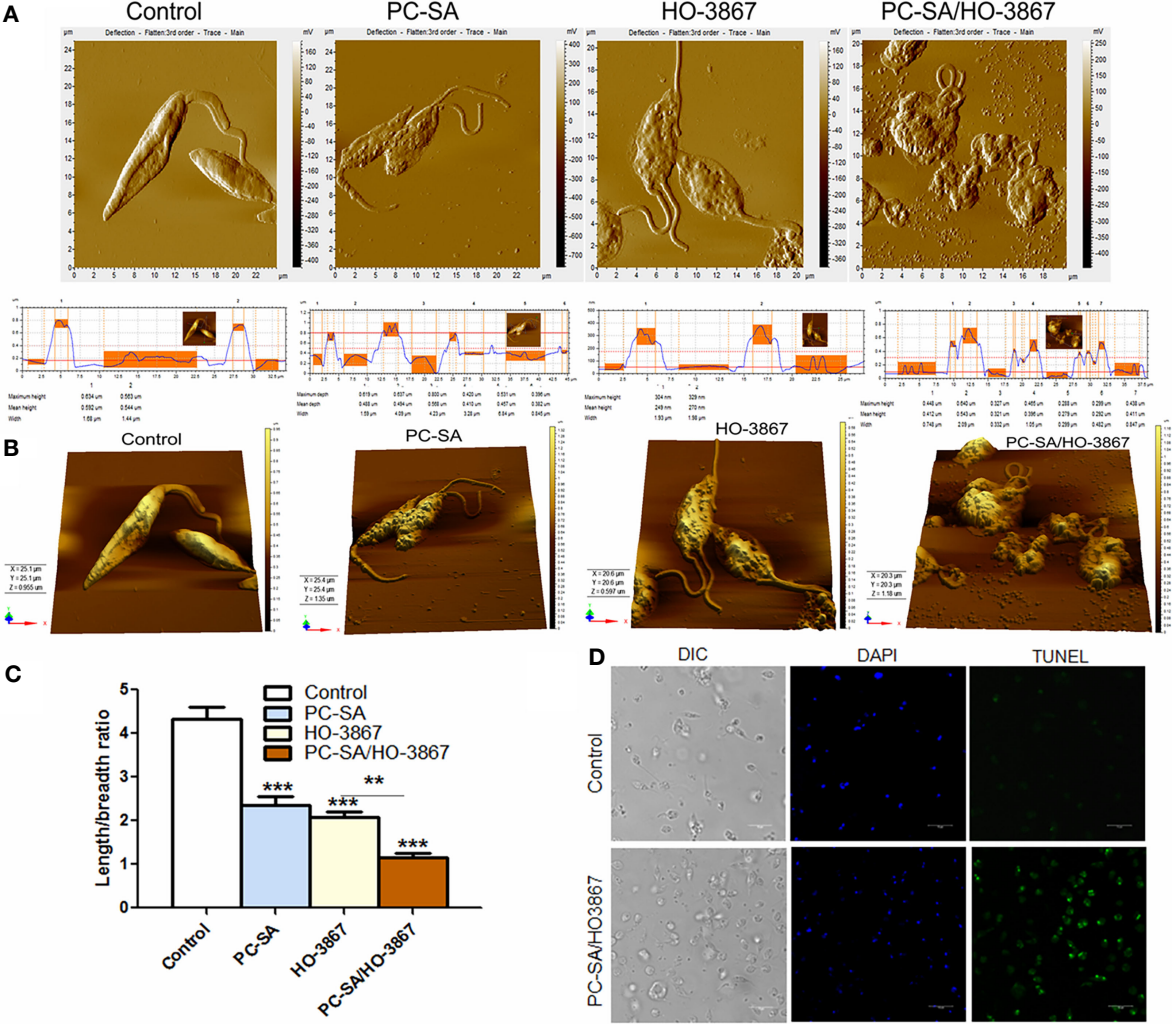
AFM images comparing the effects of free and liposomal HO-3867 upon log-phase promastigotes of *L. donovani* at half-maximal inhibitory concentration (IC_50_: 10 µg/ml or 21.5 μM). **(A)** Two-dimensional images of control, HO-3867, empty PC-SA liposomes, and PC-SA/HO-3867-treated promastigotes. Surface smoothness and height are indicated by histograms for control, liposomes, and free and liposomal HO-3867-treated promastigotes in the lower panel. **(B)** Mean body length/breadth ratio of differently treated promastigotes. Data are mean ± S.E. (n = 3) derived from one of the three independent experiments (***p < 0.001), calculated by one-way ANOVA and Tukey’s multiple-comparison test. **(C)** Representative 3D images of HO-3867, empty liposomes, and PC-SA/HO-3867-treated promastigotes after 2 h. **(D)** Confocal images showing genomic DNA fragmentation in controls and liposomal HO-3867-treated promastigotes, using TUNEL staining. Intact nuclei are visualized in blue (DAPI), and TUNEL-positive nuclei are stained green (FITC). The data represent one of the three experiments with similar results. **p < 0.01.

Marked cytoskeletal alterations like rounded cell shrinkage, decrease in flagellar length, or loss of flagella is frequently reported during PCD in *Leishmania* (Ambit et al., 2011). AFM studies revealed that both free and liposomal HO-3867-treated parasites exhibited cell shrinkage after 2 h, with maximum cytoplasmic condensation with PC-SA/HO3867 treatment (**Figures 2A, B**). Compared to the typical smooth, elongated shape of normal promastigotes, PC-SA/HO3867 treatment resulted in irregular cell morphology, membrane blebbing, and flagellar distortion (**Figures 2A–C**) in *L. donovani*. Similar morphological changes have earlier been associated with apoptosis-like cell death in *L. donovani* by clerodane diterpene (Kathuria et al., 2014) and Kalsome (Shadab et al., 2017) and curcumin in *L. infantum* (Eaton et al., 2014).

Fragmentation of nuclear DNA is an essential hallmark of apoptosis in eukaryotes. Quantification of nuclear DNA nicking in PC-SA/HO-3867-treated parasites was determined by TUNEL assay based on binding of terminal deoxynucleotidyl transferase (TdT) enzyme to the 3′-OH end of the fragmented DNA. Hence, the FITC-fluorescence obtained is directly proportional to the fragmented DNA inside cells. Promastigotes treated with 10 µg/ml of liposomal HO-3867 for 2 h showed increased DNA nicking as evidenced from enhanced green fluorescence compared to the untreated controls, observed under a confocal microscope (**Figure 2D**).

**Figure 3:**
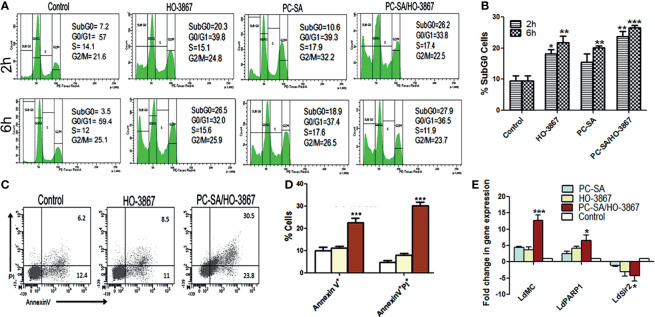
Analysis of cell cycle arrest and mechanism of cell death in free and liposomal HO-3867-treated *L. donovani* promastigotes. **(A)** Cell cycle distribution of log-phase parasites after treatment with or without liposomal HO-3867 for 2 and 6 h. **(B)** Statistical analysis of subG0 accumulation in cell cycle distribution measured by flow cytometry after free drug and PC-SA/HO-3867 treatment. **(C)** Representative flow cytometry dot plots showing promastigotes left untreated or treated with 10 µg/ml of free or PC-SA liposome-entrapped HO-3867 for 2 h were stained with Annexin V-FITC and PI. **(D)** Early (Annexin V^+^PI^-^) and late apoptosis (Annexin V^+^PI^+^) of promastigotes after Annexin V-FITC/PI staining following treatment with 10 µg/ml of free drug or PC-SA/HO-3867. **(E)** Fold change of *L. donovani* metacaspase (LdMC), LdPARP1, and LdSir2 genes relative to internal GAPDH control in drug-treated parasites compared to controls. Data (mean ± S.E.) are one of the three independent experiments. *p < 0.05, **p < 0.001, ***p < 0.0001 compared to saline control, assessed by one-way ANOVA and Tukey’s multiple-comparison test.

Targeting the cell proliferation *in vitro* can provide mechanistic insight into novel antileishmanial drugs. The effect of liposomal and free HO-3867 on cell cycle progression was examined by flow cytometry by staining the permeabilized cells with PI. PC-SA/HO-3867 induced a significant increase (~3.4-fold) in the subG0 cell cycle compared to the untreated controls after 2 and 6 h (**Figures 3A, B**). After 2 h, the percentage of cells in the subG0 phase was around 8% in the untreated promastigotes, which increased to 20% and 26%, in the *L. donovani* promastigotes treated with free and liposomal HO-3867, respectively. These data clearly demonstrate the growth-inhibitory effect of free and liposomal HO-3867 on *L. donovani*, with cell cycle arrest in the subG0 phase, observed till 6 h post-treatment.

### PC-SA/HO-3867 Induces PS-Externalization and *L. donovani* Metacaspase and PARP1 Overexpression

Cellular death has been broadly categorized into apoptotic and necrotic pathways. Apoptotic cell death in unicellular protozoan parasites is almost always associated with translocation of PS from the inner to outer leaflet of the cell membrane (Nikoletopoulou et al., 2013). Flow cytometry analysis based on Annexin V-FITC/PI dual staining was used to differentiate between apoptotic cells after treatment. Our results showed late apoptosis (Annexin V^+^PI^+^) in nearly 30% of PC-SA/HO-3867-treated cells compared to only 8.5% and 6.2% cells in free HO-3867-treated and controls, respectively (**Figures 3C, D**), without any increase in necrotic population (Annexin V^-^PI^+^). The results strongly indicate that liposomal HO-3867 treatment resulted in apoptosis of *L. donovani* largely due to increased externalization of some anionic membrane lipids like phosphatidylglycerol, phosphatidylethanolamine, and phosphatidic acid, along with a small amount of PS in the promastigote outer membrane binding Annexin V (Imbert et al., 2012; Weingärtner et al., 2012). Although *Leishmania* promastigotes possess less outer-membrane PS compared to amastigotes (Bouazizi-Ben Messaoud et al., 2017), this little amount of PS along with other anionic membrane lipids on *Leishmania* promastigotes seems to be adequate for interacting with cationic PC-SA liposomes for successful targeting of the parasites.

The viability and virulence of *Leishmania* parasites depend upon some key enzymes like metacaspase and PARP-1 (poly[ADP]-ribose polymerase-1) genes, compared to SIR2 (silent information regulator)-related proteins (Sengupta et al., 2011; Mittal et al., 2017; Basmaciyan and Casanova, 2019). Stress-induced overexpression of PARP1 can lead to suppression of sirtuins (SIR2) *via* depletion of intracellular nicotine adenine dinucleotide (NAD) levels resulting in ATP depletion, DNA damage, and cell death in protozoan parasites (Mittal et al., 2017). Real-time PCR analysis with gene-specific primers and *L. donovani* GAPDH (Purkait et al., 2012; Shadab et al., 2017) showed a 3.5- and 1.6-fold increase of *L. donovani* metacaspase and PARP1 respectively in liposomal HO-3867-treated parasites over free drugs (**Figure 3E**). Also, an almost 1.5-fold reduction in SIR2 gene expression was observed in PC-SA/HO-3867-treated parasites compared to free drugs, after 6 h. Thus, liposomal HO-3867-mediated parasite killing is associated with overexpression of *L. donovani* metacaspase, PARP1 with concomitant downregulation of SIR2 in promastigotes.

### PC-SA/HO-3867 Causes Depolarization of Mitochondrial Membrane

**Figure 4:**
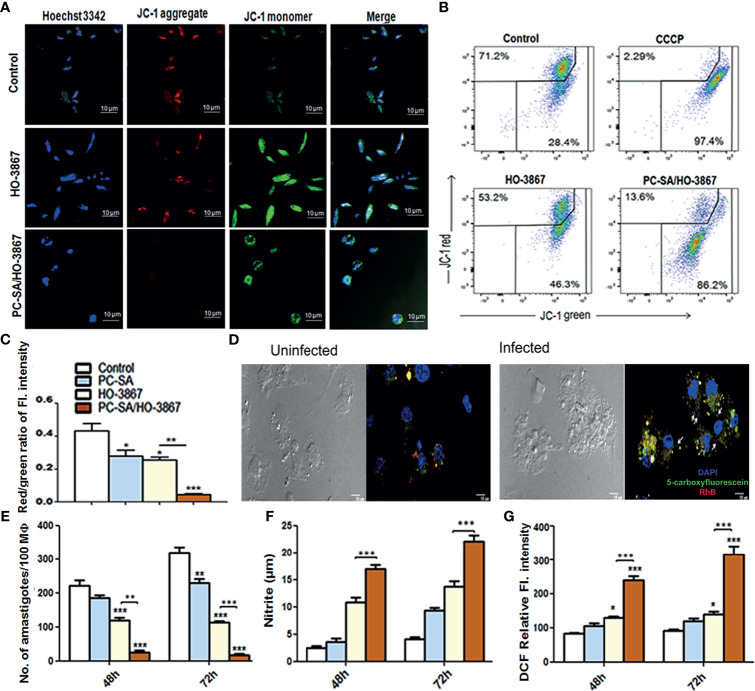
*In vitro* leishmanicidal effect of liposomal HO-3867. **(A–C)** *In situ* mitochondrial membrane potential of promastigotes after a 2-h treatment with or without liposomal HO-3867, as estimated by JC-1 staining. An almost complete JC-1 monomer was induced by 50 µM CCCP, uncoupling mitochondrial respiration, taken as positive control. Confocal images **(A)**, flow cytometry dot plot **(B)**, and the bar graph **(C)** intensity of red/green JC-1 ratio. **(D)** Internalization of fluorescent PC-SA liposomes (membrane stained with red RhB encapsulating the green 5-carboxyfluorescein stain inside core) inside the uninfected (i and *in vitro L. donovani*-infected (ii) peritoneal MΦ from healthy BALB/c mice. Intact PC-SA liposomes (yellow) are seen near macrophage nuclei (blue). White arrows indicate intracellular *L. donovani* amastigotes (small blue dots). Magnification, ×40. Data are representative of three independent experiments with similar results. **(E)** Intracellular parasite burden in peritoneal MΦ from naïve BALB/c mice infected with promastigotes treated with HO-3867 (10 µg/ml), PC-SA/HO-3867, and empty PC-SA liposomes (10 µg/ml w.r.t PC) for 48 and 72 h post infection, expressed as number of amastigotes/100 MΦs in the form of bar graph. NO **(F)** and ROS **(G)** production by the treated MΦ after 48 and 72 h in response to *in vitro* infection. Data (mean ± S.E.) are derived from the three independent experiments with similar results. *p < 0.05, **p < 0.001, ***p < 0.0001 compared to saline control as assessed by one-way ANOVA and Tukey’s multiple-comparison test.

Mitochondria are vital cell organelles for proper cellular function and viability. Disruption of mitochondrial integrity and loss of mitochondrial membrane potential are striking features of apoptosis (Lee et al., 2002; Kroemer et al., 2007). Changes in mitochondrial membrane potential in treated cells were measured by confocal microscopy and flow cytometry gating using the MitoProbe JC-1 Assay Kit (Reers et al., 1995; Shadab et al., 2017; Sivandzade et al., 2019). As shown in **Figures 4A–C**, the aggregated JC-1 (red) is released from parasite mitochondria to the cytoplasm as monomers (green) following treatment with free and liposomal HO-3867 (10 µg/ml), indicating significant mitochondrial damage after 2 h. However, in controls, intense JC-1 red fluorescence is indicative of healthy mitochondria (**Figure 4A**). The relative mitochondrial depolarization (red/green JC-1 ratio) (**Figure 4C**) showed 9.5-fold lower red/green ratios in liposomal HO-3867-treated promastigotes compared to untreated controls (**Figure 4C**).

### Internalization of PC-SA Liposomes by *L. donovani*-Infected MΦ

Macrophage (MΦ)-targeted drug delivery has been of specific interest in leishmaniasis due to the versatility of macrophages to act as host cells as well as central APCs in the processing of liposomal antigens (Kelly et al., 2011; Bogdan, 2020). A profound effect of particle size on its efficacy and biodistribution has been reported, with medium-sized liposomes of size ~150–200 nm having the longest circulation time and better efficacy (Sercombe et al., 2015; Bogdan, 2020). Confocal microscopic analysis of intracellular fates of PC-SA liposomes was carried out by labeling the vesicles with Rhodamine B (red) entrapping 5-carboxyfluorescein dye (green) (Das et al., 2018). Although no initial difference in uptake between infected and non-infected MΦ was observed after 10 min (data not shown) of incubation, more accumulation of intact PC-SA liposomes (yellow) was seen inside infected MΦ (nuclei labeled with DAPI) close to intracellular amastigotes (small blue dots) (**Figure 4D**, i and ii) compared to non-infected cells after a brief incubation time of 2 h. Interestingly, in accordance with some previous studies (Sercombe et al., 2015; Bogdan, 2020), the fluorescent PC-SA liposomes (**Figure 4D**) were seen to be preferentially internalized by the *L. donovani*-infected MΦs compared to uninfected MΦ, ensuring better antileishmanial effect of the drug *in vivo*. This is possibly due to altered phagocytosis by parasitized APCs (Noronha et al., 2000; Borborema et al., 2011). This suggests that PC-SA liposomes more effectively target parasitized MΦs than the non-infected ones, thereby increasing the efficacy of the drug.

### PC-SA/HO-3867 Activates NO and ROS Mediated Killing of *L. donovani* Amastigotes

MΦs are the main phagocytic cells playing a dual role in *Leishmania* infection acting as chief host cells sustaining amastigote multiplication as well as helping in parasite clearance (Tomiotto-Pellissier et al., 2018). To investigate whether the antileishmanial effect of PC-SA/HO-3867 observed in *L. donovani* promastigotes extended to intracellular amastigotes, *in vitro* infected murine peritoneal MΦs were treated with an IC_50_ concentration of free and PC-SA/HO-3867 (10 µg/ml) for 48 and 72 h. As shown in **Figure 4E**, a significantly higher time-dependent suppression of intracellular amastigote multiplication was noted upon PC-SA/HO-3867 treatment compared to controls and free HO-3867 at 48 h (*p* < 0.001) and 72 h (*p* < 0.0001) post-treatment in peritoneal MΦs. We further investigated the amount of secreted NO and ROS in culture supernatants of infected and drug-treated MΦs after 48 and 72 h. Compared to free HO-3867, PC-SA/HO-3867 treatment induced 1.5- and 2.6-fold higher NO (**Figure 4E**) and ROS (**Figure 4F**) generation, respectively (*p* < 0.0001), after 72 h. One plausible explanation for this enhanced efficacy of liposomal HO-3867 is the synergistic leishmanicidal effect of cationic PC-SA liposomes with HO-3867 in a synergistic manner. Collectively, our results emphasize the antileishmanial potency of liposomal HO-3867 as a strong and safe drug candidate for VL, which merit future preclinical evaluation.

## Data Availability Statement

The original contributions presented in the study are included in the article/**Supplementary Material**. Further inquiries can be directed to the corresponding author.

## Ethics Statement

The animal study was reviewed and approved by the Committee for the purpose of Control and Supervision on Animal Experiments (CPCSEA), Govt. of India, and Animal Ethics Committee (147/1999/CPSCEA) of CSIR-IICB.

## Author Contributions

AD and MK performed experiments. AD and NA equally contributed to perception and intellectual conceptualization of the work. All authors contributed to the article and approved the submitted version.

## Funding

NA is a fellow of J.C. Bose National Fellowship, which provided financial support. This work was financially supported by the Council of Scientific and Industrial Research (CSIR) grant (No. BSC0114) and UK Research and Innovation grant “A global Network for Neglected Tropical Diseases” (No. MR/P027989/1). J.C. Bose National Fellowship also for financial support, as Dr. Ali is a J.C. Bose National Fellow.

## Conflict of Interest

The authors declare that the research was conducted in the absence of any commercial or financial relationships that could be construed as a potential conflict of interest.

## Publisher’s Note

All claims expressed in this article are solely those of the authors and do not necessarily represent those of their affiliated organizations, or those of the publisher, the editors and the reviewers. Any product that may be evaluated in this article, or claim that may be made by its manufacturer, is not guaranteed or endorsed by the publisher.
