# Supplementary material for: HO-3867 Induces ROS-Dependent Stress Response and Apoptotic Cell Death in Leishmania donovani
Source: Front Cell Infect Microbiol. 2021 Dec 3;11:774899. doi: 10.3389/fcimb.2021.774899 (PMC8677699; doi:10.3389/fcimb.2021.774899)
Supplement: Supplementary file 1 [file DataSheet_1.doc]

**Supplementary material**

| Treatment | *L. donovani* promastigotes | | Intracellular amastigotes of  *L. donovani* | | % of MΦ survival at 10µg/ml (± SD) |
| --- | --- | --- | --- | --- | --- |
|  | EC_50_ (µg/ml) | EC_90_ (µg/ml) | EC_50_ (µg/ml) | EC_90_ (µg/ml) |  |
| PC-SA | 40 ± 0.11 | 53.2 ± 0.5 | 10.5 ± 1.1 | 15 ± 2.36 | 99.64 ± 3.02 |
| HO-3867 | 55 ± 2.34 | 70 ± 1.02 | 8.5 ± 0.56 | 12.2 ± 1.38 | 98.20 ± 2.84 |
| PC-SA/HO-3867 | 10 ± 0.22 | 15 ± 0.31 | 2.0 ± 0.04 | 4.25 ± 0.56 | 98.45 ± 2.62 |

**Table 1.** **Cell viability of *L. donovani* promastigotes and intracellular amastigotes inside murine MΦs cells** **RAW264.7 after treatment with empty PC-SA liposomes, free HO-3867 and PC-SA/ HO-3867, after 2h.** Promastigotes and intracellular amastigotes were treated with increasing concentrations of drug, with or without liposomes to determine the EC_50_ (µg/ml) and EC_90_ (µg/ml). Percent cell viability of uninfected RAW264.7 cells treated with 10µg/ml of liposomes, free drug and liposomal drug, for 24h was also determined. Results represent one of the two independent experiments, as mean ± S.E. (n=3).

**Figure S1:**


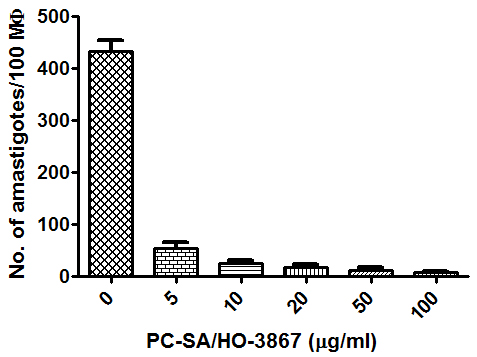


Fig. S1: **Antileishmanial potency of PC-SA/HO3867 against *L. donovani* amastigotes in RAW264.7 cells.** Murine MΦ cell line RAW264.7 (2 × 10^5^ cells/coverslip) were infected with *L. donovani* promastigotes. Infected MΦs were incubated with increasing concentrations of liposomal HO-3867 (5-100 µg/ml w.r.t PC) for 2h. Treated and non-treated MΦs were Giemsa-stained 72h post-infection and counted under light microscope. The data is represented by bar graph indicating number of amastigotes/100 MΦ. Results are indicative of mean ± S.E. of two independent experiments.
